# Epidemiological survey of mucus extravasation phenomenon at an oral pathology referral center during a 43 year period^☆^

**DOI:** 10.1016/j.bjorl.2015.09.013

**Published:** 2016-01-06

**Authors:** Thâmara Manoela Marinho Bezerra, Bárbara Vanessa de Brito Monteiro, Águida Cristina Gomes Henriques, Marianne de Vasconcelos Carvalho, Cassiano Francisco Weege Nonaka, Márcia Cristina da Costa Miguel

**Affiliations:** a Universidade Federal do Ceará (UFC), Fortaleza, CE, Brazil; b Universidade Federal de Campina Grande (UFCG), Campina Grande, PB, Brasil; c Universidade Federal da Bahia (UFBA), Salvador, BA, Brazil; d Universidade de Pernambuco (UPE), Arcoverde, PE, Brazil; e Universidade Estadual da Paraíba (UEPB), Campina Grande, PB, Brazil; f Universidade Federal do Rio Grande do Norte (UFRN), Natal, RN, Brazil

**Keywords:** Mucocele, Ranula, Minor salivary glands, Mucocele, Ranula, Glândulas salivares menores

## Abstract

**Introduction:**

Mucoceles are common benign pseudocystic lesions of the oral cavity; their main etiological factors are trauma and ductal obstruction. Two histological patterns are found: mucus retention phenomenon (MRP) and mucus extravasation phenomenon (MEP). Mucus extravasation phenomenon is the more common histological subtype and it mainly affects the lower lip. The knowledge of its main clinical features and management is important to assist health professionals in clinical practice.

**Objective:**

This study aimed to determine the relative frequency and distribution of oral mucoceles in an oral pathology reference center.

**Methods:**

Cross-sectional historical study that analyzed all cases pathologically diagnosed as mucus extravasation phenomenon by the department of anatomic pathology of an oral pathology referral center from June of 1970 to May of 2014, considering the clinical characteristics of the lesion and those relating to the patient. SPSS v. 20.0 software for Windows was used for descriptive analysis.

**Results:**

During 43 years, 719 cases of mucus extravasation phenomenon (54.7% men and 45.3% women) were registered, with the lower lip as the most commonly affected site (*n* = 484; 67.3%). The average age of patients was 20.8 years (SD ± 14.4) with a peak occurrence in the second decade of life. Most professionals had oral mucocele/ranula (*n* = 606; 84.3%) as the initial clinical impression.

**Conclusion:**

Mucus extravasation phenomenon is a lesion that primarily affects young patients, affecting mainly the lower lip, and is commonly found in oral diagnostic services.

## Resumo

**Introdução:**

Mucoceles são lesões pseudocísticas benignas comuns da cavidade oral, que possuem como principais fatores etiológicos trauma ou obstrução ductal. Dois padrões histopatológicos são encontrados: fenômeno de retenção de muco (FRM) e fenômeno de extravasamento de muco (FEM). O FEM é o subtipo histológico mais comum e tem como principal local de acometimento o lábio inferior. O conhecimento acerca de suas principais características clínicas e formas de tratamento se faz importante para auxiliar profissionais da saúde na prática clínica.

**Objetivo:**

Este trabalho objetivou determinar a frequência relativa e a distribuição das mucoceles orais em um centro de referência em patologia oral.

**Método:**

Estudo transversal de caráter histórico, no qual foram analisados os casos diagnosticados histopatologicamente como FEM pelo serviço de anatomia patológica de um centro de referência em patologia oral, no período de junho de 1970 a maio de 2014, considerando-se variáveis clínicas da lesão e relativas ao paciente. O programa estatístico *SPSS 20.0 for Windows* foi utilizado para a análise descritiva dos dados.

**Resultados:**

Durante 43 anos foram observados 719 casos de FEM (54,7% homens e 45,3% mulheres), sendo o lábio inferior (n = 484; 67,3%) o local de principal acometimento. A média de idade dos pacientes foi de 20,8 anos (DP ± 14,4), com um pico de ocorrência na segunda década de vida. A maioria dos profissionais teve mucocele oral/rânula (n = 606; 84,3%) como primeira hipótese clínica.

**Conclusão:**

O FEM é uma lesão que afeta principalmente pacientes jovens, acometendo preferencialmente o lábio inferior, sendo comumente encontrada nos serviços de diagnóstico oral.

## Introduction

Mucoceles are common benign pseudocystic lesions of the oral cavity that develop secondary to leakage or retention of mucous material from salivary glands, principally the minor salivary glands.^1^ These lesions account for 70% of cystic and pseudocystic lesions of the salivary glands, thus they can appear anywhere on the oral mucosa where there is a salivary gland.^1^, ^2^

Etiologic factors involved in mucocele formation include trauma and ductal obstrction,^3^, ^4^ which may lead to formation of two histological presentations: the phenomenon of mucus retention or extravasation.^1^, ^5^ The mucus extravasation phenomena (MEP) are actually pseudocysts, as they are devoid of epithelial lining. The extravasated mucus induces an inflammatory and granular reaction in an attempt to contain the extravasation.^4^, ^6^ This type of mucocele is commonly found in minor salivary glands.^7^ The mucus retention phenomenon (MRP) induces formation of another type of mucocele: the mucus retention cyst or sialocyst.^1^ Most of these grow from major salivary glands and ducts and have the following etiological factors: sialolithiasis, periductal scarring, or invasive tumors.^5^, ^7^ Histopathologically, MRP are characterized by epithelium lining coming from the partially obstructed salivary duct.^4^

Clinically, there is no difference between the mucus extravasation and retention phenomena, and both present with increased volume of cystic appearance, are painless, soft to palpation, and show a transparent bluish color.^2^ The most common site of extravasation mucoceles is the lower lip, which is also the most susceptible place to injuries. Such location is less common in retention mucoceles,^2^, ^5^ which may affect any other site of the oral cavity.^8^ Extravasation mucoceles affect young patients (20–30 years) and usually resolve spontaneously, requiring surgical excision in some cases.^2^ Micro-marsupialization, cryosurgery, steroid injections, and CO_2_ laser have been described as treatments available for these lesions.^2^, ^5^, ^8^, ^9^

The aim of this study was to perform an epidemiological survey of oral mucocele cases diagnosed in the Department of Anatomic Pathology of the Oral Pathology Discipline of a university in the period of 1971–2014, as well as to describe the main clinical characteristics and treatment, in order to assist health professionals in clinical practice.

## Methods

Cross-sectional, retrospective, historical study, approved by the Research Ethics Committee under No. #675,422, that included 719 cases of oral mucoceles selected from 11,589 cases recorded in the files of the Pathologic Anatomy Laboratory between June of 1970 and May of 2014.

The collection procedure was based on the information in the histopathology reports and medical records of biopsies, which were transcribed into a standard file developed specifically for this analysis. The assessed variables related to the patient were gender, age, and race. Regarding clinical features of oral mucocele, the following data were collected: lesion location, shape of the main lesion, surface, symptoms, duration, size, history of trauma, size variation, clinical diagnosis, and type of biopsy performed. It is noteworthy that because ranula is considered a clinical variant of mucoceles,^8^ it was decided to group them together in this study.

## Results

The 719 cases of oral mucoceles represent 5.8% of all cases registered by the oral pathology laboratory during the stated period. Considering the anatomical site, it was found that the lower lip was the most affected region (67.3%; Table 1). Regarding patients’ gender, the lesion was more frequent in females (54.7%). The average age of patients with mucocele was 20.8 years (SD ± 14.4), with a range of 1–82 years old. A higher prevalence of lesions was observed in white patients (48.4%; Table 2).

**Table 1:** Distribution of absolute and relative frequencies regarding the location of oral mucoceles.

Anatomic location	Number (*n*)	Percentage (%)
Lower lip	484	67.3
Upper lip	2	0.3
Palate	4	0.6
Cheek mucosa	27	3.8
Tongue	58	8.1
Floor	69	9.6
Others	60	8.3
No information	15	2.1
Total	719	100

**Table 2:** Distribution of absolute and relative frequencies according to skin color of patients affected by oral mucocele.

Skin color	Number (*n*)	Percentage (%)
Leucoderma	348	48.4
Melanoderma	135	18.8
Feoderma	133	18.5
No information	103	14.3
Total	719	100

The most frequent clinical aspects of oral mucoceles were a nodular (59.8%) asymptomatic (70.9%) lesion, with a smooth surface (13.1%), measuring on average 0.9 cm (SD ± 0.70), with the same color as the mucous membrane (36.2%), and mean duration of 20 weeks (SD ± 28.7), ranging from one week to six years (Table 3). Regarding the medical history, some patients reported previous trauma to the formation of the lesion (23.6%) and only 7.2% reported at the time of the interview that they noticed the lesion showing variation in size during the clinical course (Table 3). Upon first contact with the lesion, most dentists listed oral mucocele/ranula (84.3%) as their first clinical impression (Table 4). Ultimately, excisional biopsy was the treatment of choice for most lesions (83.3%).

**Table 3:** Distribution of absolute and relative frequencies regarding clinical features of oral mucoceles.

Clinical features	Variable	Number (*n*)	Percentage (%)
Surface	Smooth	94	13.1
	Rough	17	2.4
	Ulcerated	3	0.4
	No information	605	84.1

Color	Whitish	97	13.5
	Reddish	86	12
	Yellowish	13	1.8
	Bluish/Purplish	75	10.4
	Translucent	66	9.2
	Similar to the mucosa	260	36.2
	Others	27	3.8
	No information	95	13.2

Type of lesion	Polypoid	52	7.2
	Bulging mass	430	59.8
	Blister	14	1.9
	No information	223	31

Symptoms	Symptomatic	51	7.1
	Asymptomatic	510	70.9
	No information	158	22

Size range	0–2 cm	622	86.5
	2.1–4 cm	27	3.8
	From 4.1 cm	2	0.3
	No information	68	9.5

Age group	0–10 years	163	22.7
	11–20 years	264	36.7
	21–30 years	139	19.3
	31–40 years	59	8.2
	41–50 years	19	2.6
	51–60 years	15	2.1
	61–70 years	15	2.1
	71–80 years	5	0.7
	81–90 years	2	0.3
	No information	38	5.3

History of trauma	Yes	170	23.6
	No	48	6.7
	No information	501	69.7

History of volume change	Yes	52	7.2
	No information	667	92.8

**Table 4:** Distribution of absolute and relative frequencies regarding the main clinical hypothesis of oral mucocele.

Clinical diagnosis	Number (*n*)	Percentage (%)
Mucocele/ranula	606	84.3
Inflammatory fibrous hyperplasia	28	3.9
Fibroid	26	3.6
Papiloma	12	1.7
Mucus retention cyst	8	1.1
Lipoma	6	0.8
Hemangioma	4	0.6
Pyogenic granuloma	2	0.3
Other	10	1.4
No information	17	2.4
Total	719	100

## Discussion

The incidence of mucocele is generally high in the population, with 2.5 lesions per 1000 patients.^2^ This injury was the 17th most common affecting the oral cavity, representing 0.08% of oral lesions in a study in Brazil.^10^ The term mucocele is strictly clinical and serves to describe increased volume caused by saliva coming from broken or clogged duct of minor salivary gland. Oral mucoceles located in the floor of the mouth are called ranula and commonly originate from the body of the sublingual gland, and occasionally from Rivini's duct or Wharthon's duct.^11^

Among the benign lesions of minor salivary glands, mucocele is the most common.^8^, ^12^, ^13^ Cecconi et al.^5^ found that mucocele accounts for 4.61% of oral cavity biopsies, indicating that it is a lesion commonly found in oral medicine services. Similarly, a 15-year retrospective study by Mohan et al.^14^ found 393 salivary gland lesions, of which 216 were reported as non-neoplastic lesions, with mucocele being the most prevalent lesion (54.5%). These findings are similar to data from this study, where oral mucocele accounted for 5.8% of all lesions diagnosed during 44 years of the Pathologic Anatomy Laboratory service of the Department of Dentistry of a university.

Minor salivary glands are found anywhere in the mouth, except the gums.^8^ Thus, retention and extravasation mucoceles are commonly found in different locations, with no clinical differences between them.^2^, ^15^, ^16^ To Bhargava et al.,^6^ histopathological examination is crucial to confirm the clinical diagnosis. Microscopically, mucous extravasation phenomena are pseudocysts because they have no epithelial wall covering, and occur in three evolutionary phases. First, there is mucin spill into the surrounding tissue, where some leukocytes and histiocytes are seen. Second, the granulation reaction appears, with the presence of histiocytes, macrophages, and multinucleated giant cells associated with a foreign body reaction. Later, in the third stage, there is the formation of a pseudocapsule without epithelium around the mucosa, comprised of connective tissue cells.^2^ The retention cysts characteristically present with a cystic cavity lined by cuboidal epithelium and exhibit less inflammatory reaction.^6^ According to Bhargava et al.,^6^ during surgery, when the fibrous wall of the lesion appears thick, it may be a salivary gland neoplasm. However, regardless of the lesion wall thickness and characteristic clinical findings, it is recommended to send a portion for histopathological examination to confirm the clinical suspicion.

Extravasation mucoceles are the most common subtype^8^, ^13^ and often appear in the lower lip.^2^ However, the retention-type mucoceles are rarely found at that location,^5^ and may appear elsewhere in the oral cavity,^2^ as they are more closely related to major salivary glands.^4^, ^6^ The most frequently affected sites for retention mucoceles in the minor salivary glands are the upper lip, palate, cheeks, oral floor, and maxillary sinus.^5^, ^8^ In the present study, all cases analyzed were extravasation mucocele.

Among the 719 mucoceles analyzed in this study, the majority (67.2%) occurred in the lower lip. Several published epidemiological studies validate this finding.^2^, ^5^, ^10^, ^12^, ^13^ The lower lip is the anatomic site in the oral cavity most prone to trauma, especially in the premolar region.^2^, ^17^ This fact explains the high incidence of extravasation mucoceles in this region, as trauma is suggested as the primary etiologic factor of these lesions.^5^, ^14^ To Khanna et al.,^18^ parafunctional habits, such as biting and sucking the lower lip, are also related to the higher incidence in this region, but such movements ultimately lead to local traumas that converge to a single etiologic factor. A survey of 2788 cases of lesions resulting from trauma showed that the lower lip is not only the most frequent area for mucocele occurrence (*n* = 676; 64.5%), but also the most affected site of all soft tissue injuries associated with trauma (*n* = 815; 29.5%).^12^ Although it was not possible in this study to collect information regarding history of trauma in 69.7% of biopsy records, a considerable proportion of patients (23.6%) reported history of previous trauma before the lesion formation. Trauma is the etiological factor most well documented and accepted in studies of oral mucoceles.^6^

According to some studies in the literature, there is no difference in the incidence of oral mucoceles between genders,^6^, ^17^, ^19^ similar to the present study. However, in other studies, the incidence of mucocele between genders is quite variable, with cases of higher prevalence both among women^20^ and among men.^10^, ^12^, ^16^ According to an Indian study, men in the second decade of life are most affected by mucocele due to many psychological problems, leading them to the habit of biting the lip, which would cause injury.^21^ Despite these differences between studies, they all recognize that trauma is the main etiological agent for this type of injury, with variations between the sexes on the trauma origin (stress, psychological factor, bite, etc.).

Extravasation mucoceles affect patients of all ages, with a high incidence in the second decade of life.^2^, ^6^, ^8^, ^10^, ^12^, ^19^, ^20^ In contrast, MRP commonly affect older individuals.^1^, ^4^, ^20^ In agreement with the literature, the age group most affected in this study was the second decade of life (Table 3). Furthermore, a case of mucocele in a one-year-old child was found. Mucoceles can be congenital or arise immediately after birth, but they are rare in children under one year of age.^6^, ^13^

Clinical presentation of oral mucocele may vary depending on the lesion depth.^10^ Those located just below the mucosa present superficially with the clinical features of a vesicle or blister; those located in the upper submucosa comprise the classic mucoceles, with the clinical feature of a nodule.^8^ These variations are seen in detail in Table 3. The color of mucoceles varies between mucosal color (pink) to bluish, and the depth of tissue damage also has an influence in this regard.^5^, ^7^ Superficial lesions cause tissue stretch, which makes the tissue thinner and cyanotic and causes vascular congestion, resulting in bluish lesions.^2^, ^5^, ^8^ However, deeper mucoceles are well-circumscribed masses covered by normal oral mucosa.^8^ The size of the lesion and the elasticity of its lining tissue are other factors that influence this clinical aspect.^7^, ^21^ In the present study, oral mucoceles simulated these findings (Table 3).

Despite the clinical variations of mucoceles identified in this study, they are small and their appearance is pathognomonic:^8^ nodular lesion; asymptomatic; smooth surface; and with a variety of colors, such as blue, transparent, pink, and whitish.^2^, ^5^, ^6^ The lesions may be solitary or multiple, and do not have constant duration, ranging from a few days to three years.^8^ Thus, despite mucoceles being a clinical diagnosis,^2^, ^8^, ^10^ it usually is not difficult for the dentist to identify them, which facilitates the targeted treatment. These findings can be observed in this study where most professionals (78.4%) indicated mucocele as the initial clinical impression, which resulted in the excisional biopsy as the primary treatment of choice (83.4%). Thus, the patient's medical history combined with information about the location, history of trauma, rapid onset, change in size, lesion color and consistency lead to the correct diagnosis of the lesion and clinical management by the professional.^6^ It is noteworthy that some specific cases may require further evaluation for diagnosis, such as routine X-rays, ultrasound, or methods of advanced diagnostics (computed tomography and magnetic resonance imaging) in order to better visualize the shape, diameter, position, and to determine the source of the lesion.^1^, ^8^ The cytological technique of fine-needle aspiration is a useful tool for the diagnosis of patients with nodules and masses in the salivary glands, especially when considering angiomatous lesions in the differential diagnosis.^1^, ^2^

In most cases, although the clinical diagnosis of mucocele does not offer difficulties for the professional, its similarity to other lesions that can affect the oral cavity is common. Superficial mucocele may be confused with scar pemphigoid, bullous lichen planus, or herpes.^22^, ^23^ The differentially diagnosis of deeper mucoceles includes lipoma, hemangioma, oral lymphangioma, benign and malignant tumors of salivary gland origin, varicose vein, irritation fibroma, oral lymph-epithelial cyst, gingival cyst of the adult, tissue abscess, cysticercosis, pyogenic granuloma, and others.^8^

It is noteworthy that some superficial mucoceles with a history of recurrence have been associated with oral lichen planus and chronic graft versus host disease.^22^, ^23^ To Bermejo et al.,^22^ the patient's oral mucosa with erosive lichen planus undergoes constant erosion and re-epithelialization, enabling the rupture of a small output of the salivary gland duct, which causes mucus accumulation below the epithelium. There is also the possibility that the inflammatory process may play a role in the pathogenesis of recurrent mucoceles, as lymphocytic infiltrate can block the duct of accessory gland, which would induce its disruption and subepithelial extravasation of mucus.^23^ The correct identification of recurrent superficial mucocele and other conditions that may be associated with it contribute to a better clinical management of patients.

Clinical findings of paramount importance are often not transcribed into biopsy record, which undermines any epidemiological study. In the present study, the variables “history of trauma” and “history of volume change” were not present in biopsy records, which are structured, and added to freely by dentists or graduate students. The variable “history of volume change” was indicated as present in only 7.2% of biopsy records. It is believed that this percentage does not correspond to the reality of this finding, as much of the biopsy records are structured, there is no room for additional information that may be relevant not only to the correct histopathological diagnosis but also for future epidemiological studies that use these records as tools. Furthermore, a variation in size is a very characteristic clinical feature of oral mucoceles. According to Cecconi et al.^5^ it occurs due to rupture, especially in superficial lesions, or reabsorption of saliva and subsequent accumulation of mucus.^5^, ^8^ Likewise, the occurrence of a trauma in the wound region was recorded in only 23.6% of cases, while 69.7% did not add this information (Table 3). Similarly to the previous data, it does not match the expected, and may also be treated, in this case, as absence of information. A history of trauma is widely reported in the literature as the main cause of extravasation mucocele.^8^

It was found that most oral mucoceles (*n* = 510; 70.9%) were asymptomatic, as only 7.1% (*n* = 51) presented some symptoms (Table 3). A clinicopathological study of 25 oral mucoceles by Bagán Sebastian et al.^24^ found that only 4% of patients had some discomfort, but not pain. According to Baurmash,^7^ mucoceles greater than 1.5 cm are located more deeply in the tissues. Thus, minor lesions bring less discomfort to the patient because they are more superficial, and are located in less vascularized tissue layers and less rich in nerve structures. In addition, large lesions may impair speaking or chewing.^5^, ^7^ In the present study, on average, patients with oral mucoleles took four months to seek treatment, and some exhibited five years of evolution. According to Bhargava et al.,^6^ because there are no painful symptoms, it is usually the professional who detects the lesion in a routine oral examination.

There are no differences in the management of retention and extravasation mucoceles, and surgical removal is the standard method widely used for both.^2^, ^6^ According to Romeo et al.,^25^ surgical excision is the only treatment for this lesion, as constant relapses in extravasation mucocele are observed when this procedure is not performed. In the present study, excisional biopsy was the most widely used form of treatment for extravasation mucocele, which is consistent with the recommendations in the literature.^1^, ^2^, ^4^, ^5^, ^6^, ^7^, ^8^, ^10^, ^13^, ^20^ A strategy to prevent recurrences is the excision of small lesions down to the muscular plane, together with a margin of salivary gland tissue. In case of large lesions, marsupialization help prevent damage to vital structures, such as the labial branch of the mental nerve.^17^ Micro-marsupialization can be considered as an alternative surgical method in case of pediatric patients, as it is a simple technique, less traumatic, relatively painless, and has a low likelihood of recurrence.^6^, ^17^

Another surgical method of oral mucocele management is ablation with CO_2_ laser, which decreases the chances of recurrence and complication and allows fast and simple lesion ablation. This procedure is also suitable for patients who cannot tolerate long procedures.^17^ This technique has the disadvantage of not allowing histological examination of the lesion.

## Conclusions

Oral mucocele is a fairly common lesion that primarily affects young patients and preferentially affects the lower lip. Extravasation mucocele is the most common subtype, affecting patients. Conservative surgery is the most widely used treatment, but alternative forms have been reported in the literature, such as marsupialization, dissection, cryosurgery, carbon dioxide laser, electrocauterization, intralesional injection of sclerosing agents, and steroid injections.

This study has limitations, as do all retrospective studies. It was not possible to determine some clinical data due to lack of correct completion of biopsy records. The correct handling of this document is an important learning tool for students, as an epidemiological research tool as well as a tool for patient monitoring. We emphasize the importance of health professionals and students being stricter not only with the patient's medical history, but also in better completion of clinical records.

## Conflicts of interest

The authors declare no conflicts of interest.
